# Enhancing Asphalt Performance with CR/SBS Pellet: A Multiscale Investigation from Performance Characterization to Modification Mechanism

**DOI:** 10.3390/polym18121474

**Published:** 2026-06-12

**Authors:** Wen Li, Zenggang Zhao, Wei Li, Weiwen Quan, Dawei Dong, Shuyang Chen, Shaopeng Wu

**Affiliations:** 1 School of Civil Engineering, Central South University of Forestry & Technology, Changsha 410004, China; t20222710@csuft.edu.cn (W.L.); lwlh_fyy@csuft.edu.cn (W.L.); quanweiwen@csuft.edu.cn (W.Q.);; 2 Beijing Lude Yongtai Environmental Protection Technology Co., Ltd., Beijing 101300, China; 3 State Key Laboratory of Silicate Materials for Architectures, Wuhan University of Technology, Wuhan 430070, China

**Keywords:** polymerized pellets, modified asphalt, physical properties, rheological properties, modification mechanism

## Abstract

The emergence of a novel crumb rubber (CR)/SBS-polymerized pellet has simplified the complex preparation process of composite-modified asphalt. However, the effectiveness of CR/SBS-polymerized pellets in improving asphalt performance has not been confirmed. This study mainly investigated the performance and reinforcement mechanism of polymerized pellet-modified asphalt. First, polymerized pellet-modified asphalt samples with different contents (10%, 20%, 30% and 40% of the asphalt mass) were prepared. Then, the physical properties, rheological behavior, thermal stability, and aging resistance of the pellet-modified asphalt samples were systematically evaluated, using both base asphalt and a commercially available styrene–butadiene–styrene triblock copolymer (SBS)-modified asphalt as control groups for comparison. Finally, the modification mechanism was explored through Fourier transform infrared spectroscopy (FTIR) and fluorescence microscopy (FM). The findings demonstrated that the incorporation of polymerized pellets could effectively decrease the penetration, elevate the softening point, and enhance the viscosity of asphalt. In addition, the high- and low-temperature performance, as well as the aging resistance of the modified asphalt, were significantly improved. These enhancing effects became more pronounced with increasing modifier content. The performance of SBS-modified asphalt is between 20% pellets MA and 30% pellets MA. The pyrolysis temperature range of all asphalt samples is 220 °C~500 °C, and infrared spectroscopy indicated that CR/SBS pellet-modified asphalt is mainly a physical mixing process. This work provides a scientific basis for further engineering applications of CR/SBS pellets.

## 1. Introduction

The performance requirements for asphalt pavement materials in road engineering have become increasingly stringent, driven by the rapid growth of highway traffic volume and the continual elevation of construction standards [1]. To meet the requirements of heavy load, long service life, and low maintenance, polymer-modified asphalt has become a key material for improving road durability due to its excellent mechanical properties and environmental adaptability [2,3]. Research has demonstrated that employing polymer-modified asphalt in the production of asphalt mixtures can significantly mitigate pavement distress, such as cracking and rutting, thereby extending the service life of the pavement [4,5].

At present, commonly used polymer modifiers in asphalt include rubber, resin, thermoplastic elastomers and others, among which rubber modifiers and thermoplastic elastomers are widely researched and applied worldwide [1,6,7,8]. The generation of waste tires has risen sharply in recent years [9,10]. Improper disposal of waste tires can severely damage the ecological environment [11]. For example, outdoor stacking can easily breed mosquitoes and insects and increase the risk of fires [12,13]. The use of crumb rubber (CR) derived from waste tires as an asphalt modifier has been extensively investigated in research and widely implemented in engineering practice. Extensive research has confirmed that the incorporation of crumb rubber (CR) into asphalt can substantially enhance its key performance characteristics, including high-temperature stability, low-temperature crack resistance, and fatigue life [14]. Nevertheless, CR modified asphalt is also associated with several drawbacks, such as insufficient storage stability and challenges related to construction workability [15,16]. SBS is one of the most widely used thermoplastic elastomer modifiers [17,18]. Research has shown that SBS modifiers can markedly enhance asphalt performance, including improved high and low temperature properties, increased elasticity, and greater fatigue resistance, leading to extended pavement service life [19,20,21]. However, SBS-modified asphalt also has disadvantages such as high cost and susceptibility to aging [20,22]. In summary, a single modifier can improve one or more properties of asphalt, but there are also certain drawbacks. Consequently, composite modification utilizing multiple modifiers has been developed to enhance the overall performance of asphalt.

Multivariate composite modification technology has emerged as a significant research and application focus within the asphalt materials field [23,24]. This technology aims to overcome the limitations of a single modification system by compounding two or more modifiers (such as different polymers, nanomaterials, stabilizers, etc.), utilizing the complementary advantages of each component to enhance the comprehensive performance of asphalt materials [25,26]. In response to the respective advantages and disadvantages of rubber modifiers and SBS modifiers, the composite modification of asphalt using CR and SBS has attracted considerable research attention. Guo et al. [27] employed a central composite design combined with response surface methodology to formulate and optimize the material composition of compatibilized SBS/CR composite-modified asphalt, and evaluated its physical properties, rheological properties, and functional group changes. Research has shown that the optimized material composition is 18.2% CR, 5.2% SBS, and 17.1% solubilizer. In addition, it has been found that the performance of compatibilized SBS/CR composite-modified asphalt is better than that of asphalt modified solely with CR, solely with SBS, and their two composite-modified asphalts. Yalikun et al. [28] investigated the preparation process and performance of oxidized CR/SBS composite-modified asphalt. The research demonstrated that the incorporation of catalytically oxidized CR together with SBS markedly enhanced both the high and low temperature properties of the asphalt, and that the resulting performance exceeded that of conventional CR/SBS composite-modified asphalt. Li et al. [29] optimized the material composition of fast-melting SBS (F-SBS) and CR composite-modified asphalt and evaluated its performance. Research has shown that 3% F-SBS and 4% CR, as well as 1% F-SBS and 8% CR, both exhibit better high-temperature and low-temperature performance. In addition, the composite modification of asphalt using these two modifiers has been determined to occur primarily through a physical blending process. Xu et al. [30] reported that the low-temperature crack resistance of asphalt can be significantly enhanced through modification with high-solubility rubber combined with SBS. Song et al. [31] demonstrated that asphalt modified with a composite of 13% CR and 2% SBS exhibits better high- and low-temperature performance than asphalt modified with 5% SBS alone. The above research generally believes that CR/SBS composite-modified asphalt is considered an economically efficient and high-performance green road engineering material. However, composite modification technology not only requires precise control of the dosage of each component, but sometimes also needs to follow a specific addition sequence, making the operation process more complicated. Recently, Beijing Lude Yongtai Environmental Protection Technology Co., Ltd. has developed a polymerized pellet mainly composed of activated CR and SBS. Polymerized pellets are considered a promising material for enhancing asphalt pavement performance. However, their specific effects on asphalt properties and the underlying modification mechanisms remain insufficiently studied.

**Figure 1:**
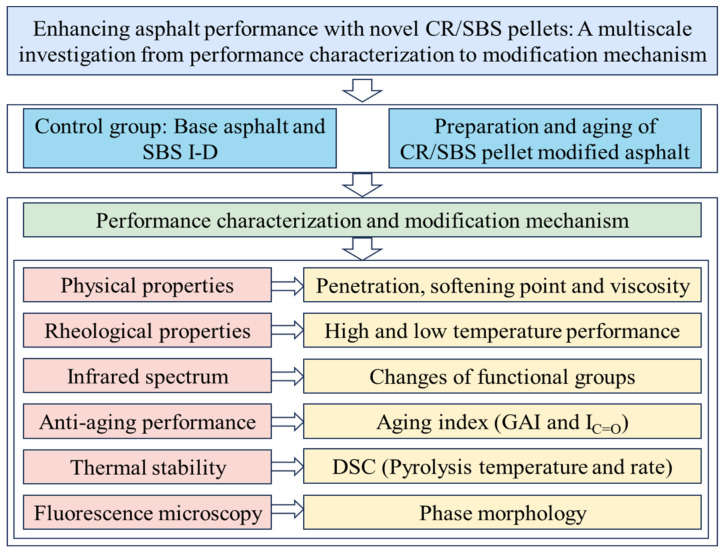
The overall research procedure of this study.

This study employs a novel CR/SBS pellet to prepare modified asphalt and systematically evaluates its performance and modification mechanism. First, modified asphalt samples with different dosages (10%, 20%, 30% and 40% of the asphalt mass) of CR/SBS pellets were prepared. Subsequently, both base asphalt and a commercially available SBS-modified asphalt (SBS I-D) were employed as control groups for performance comparison; the physical characteristics, rheological behavior, aging resistance and thermal stability of modified asphalt with CR/SBS pellets were investigated. Finally, the modification mechanism was elucidated from the perspectives of chemical structure and micromorphology through FTIR and FM. The overall research procedure is outlined in Figure 1.

## 2. Materials and Experiments

### 2.1. Raw Materials

In this study, 70-penetration-grade petroleum asphalt was used as the base asphalt, and a commercial SBS-modified asphalt (SBS I-D) served as the control. The conventional technical properties of both asphalt types were tested, with the results summarized in Table 1.

**Figure 2:**
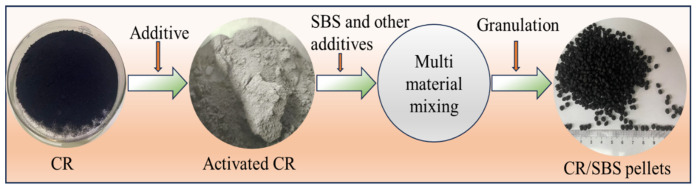
The preparation process diagram of CR/SBS pellets.

The CR/SBS pellets are provided by Beijing Lude Yongtai Environmental Protection Technology Co., Ltd. (Beijing, China). The preparation process involves first desulfurizing and activating the waste tire rubber powder, then adding SBS and other modifiers for multi-material mixing, and, finally, using a water ring granulator for granulation (Jiangsu Lvjinren Rubber and Plastic Technology Co., Ltd. (Suqian, China); Screw Extruder: LJR-75-500). Finally, ventilate and dry before packaging. The pellets are composed of 90% rubber powder (average particle size about 0.3 mm), 6% SBS, and other auxiliary additives, with a diameter of about 3 mm. Figure 2 illustrates the process of preparing polymerized pellets.

### 2.2. Experiments

#### 2.2.1. Preparation and Aging of Asphalt Modified with CR/SBS Pellets

**Figure 3:**
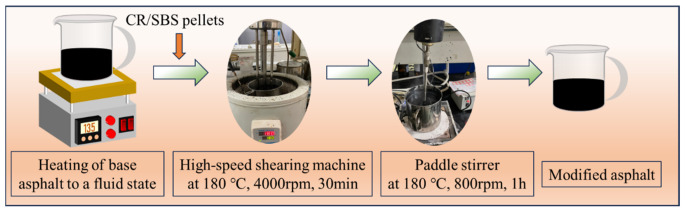
The preparation procedure for CR/SBS pellet-modified asphalt.

This study selected CR/SBS pellet dosages of 10%, 20%, 30%, and 40% of asphalt mass, respectively. Preparation method: (1) base asphalt was heated in an oven at 135 °C to achieve and maintain a fluid state, (2) add the weighed pellets modifier to the base asphalt, and the asphalt was subjected to high-speed shearing at 4000 rpm and 180 °C for 30 min, and (3) modified asphalt samples were obtained by stirring at 800 rpm at 180 °C for 1 h using a paddle mixer [32]. The modified asphalt samples prepared with four pellet modifier contents were designated as 10% pellets MA, 20% pellets MA, 30% pellets MA, and 40% pellets MA, respectively. The preparation procedure for CR/SBS pellet-modified asphalt is illustrated in Figure 3.

To assess the aging resistance of the modified asphalt, first pour about 50 g asphalt into a stainless steel disk, then place the aging disk in a film oven at 163 °C and 5.5 rpm maintain for 5 h to obtain short-term aging asphalt, and, finally, place the short-term aging asphalt in a pressure aging tester and maintain for 20 h at 2.1 MPa and 100 °C to obtain long-term aging asphalt.

#### 2.2.2. Physical Properties Tests

Physical properties tests are conducted on base asphalt, modified asphalt with different dosages, and SBS I-D, including penetration, softening point, and rotational viscosity tests. The temperature gradients for rotational viscosity testing are 120 °C, 135 °C, 150 °C, 165 °C, and 180 °C, respectively.

#### 2.2.3. Rheological Properties Tests

Asphalt exhibits typical viscoelastic behavior, and its rheological properties are significantly affected by temperature [33,34]. Temperature sweep tests were carried out on base asphalt, modified asphalt with four different CR/SBS pellet contents, and SBS I-D using a dynamic shear rheometer (DSR, MCR101, Anton Paar, Sydney, Australia). The temperature range in the temperature sweep tests is 30~80 °C, with a loading frequency of 10 rad/s. The strain control mode is used, and the shear strain of unaged asphalt is selected as 12%, while the shear strain of asphalt samples after PAV aging is selected as 1%.

A bending beam rheometer (TE-BBR, Shanghai, China) was used to evaluate the low-temperature rheological properties of base asphalt, modified asphalt with pellet modifiers, and SBS I-D. Low-temperature performance was assessed at three temperatures, −12 °C, −18 °C, and −24 °C, using creep stiffness and creep rate measured at 60 s as key evaluation parameters.

#### 2.2.4. FTIR Test

FTIR was employed to analyze the functional groups of the modifier pellets, base asphalt, pellet-modified asphalt, and SBS I-D, in order to examine functional group changes induced by the modification process. In order to avoid the influence of the dissolution process on the infrared spectrum results of modifier pellets, total reflection infrared spectroscopy was used for pellet modifiers, and the transmission method was used for detecting asphalt and modified asphalt.

#### 2.2.5. FM Tests

**Figure 4:**
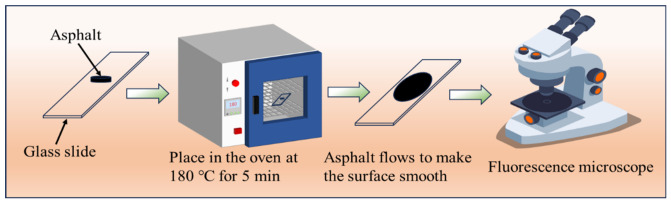
The detailed preparation process of the test samples.

Based on the difference in fluorescence behavior between asphalt and modifier under monochromatic excitation light, the dispersion state of modifier was characterized by fluorescence microscopy imaging technology. The sample was prepared by the hot casting method. The detailed procedure for preparing the test specimens is presented in Figure 4.

#### 2.2.6. Thermal Stability Tests

Differential scanning calorimetry (DSC) was used to test the thermal properties of modifier pellets, base asphalt, pellet-modified asphalt, and SBS I-D. All tests were performed in a nitrogen environment using a sample mass of approximately 10 mg. The temperature was increased from 25 °C to 1000 °C.

## 3. Results and Discussion

### 3.1. Conventional Physical Properties

**Figure 5:**
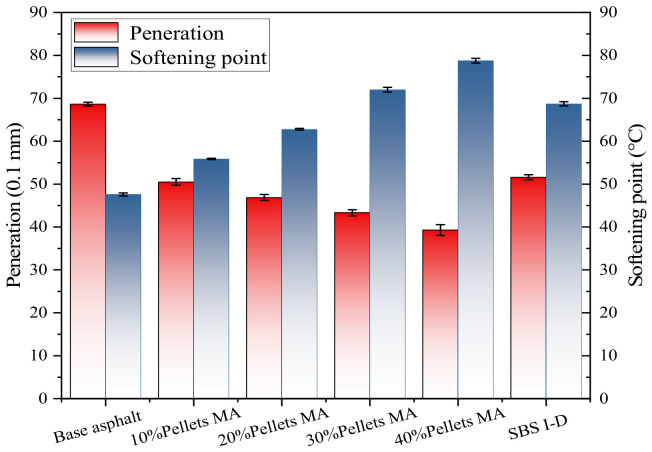
Penetration and softening point of asphalt samples.

Penetration and softening point are important indicators for characterizing consistency and high-temperature performance of asphalt [35]. A lower penetration value indicates higher consistency of asphalt, while an elevated softening point reflects improved high-temperature stability. The results of penetration and softening point tests for the base asphalt, modified asphalt with CR/SBS pellets, and SBS I-D are presented in Figure 5. The results show that incorporating modifier pellets markedly reduces the penetration and raises the softening point of asphalt. Furthermore, both effects become more pronounced as the pellet content increases. That is to say, adding modifier pellets can increase the consistency of asphalt and improve its high-temperature stability. In addition, the penetration of the 10% pellets MA was found to be comparable to that of SBS I-D, while the softening point of SBS I-D fell between the values obtained for the 20% pellets MA and 30% pellets MA.

**Figure 6:**
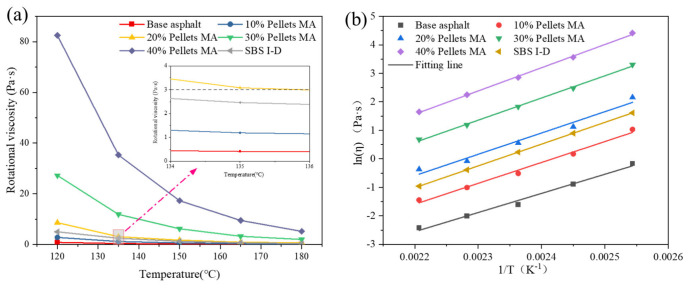
(**a**) Viscosity−temperature relationship curve; (**b**) the straight line of viscosity−temperature fitting.

The rotational viscosity of asphalt refers to the resistance of asphalt samples to rotating rotors under specific conditions, reflecting its flow characteristics and high-temperature performance, which in turn influences workability during construction [36]. Figure 6a illustrates the rotational viscosity of base asphalt, modified asphalt with four different amounts of modifier pellets, and SBS I-D at different temperatures. As temperature rises, the viscosity of all asphalt samples progressively declines, similar to an exponential decay function. Furthermore, the viscosity of the modified asphalt increases progressively with higher CR/SBS pellet modifier content. To ensure sufficient workability, the Strategic Highway Research Program (SHRP) specifies that the viscosity of modified asphalt at 135 °C should remain below 3 Pa·s [37]. The results indicate that at a CR/SBS pellet content of 20%, the viscosity of the modified asphalt approximates the 3 Pa·s threshold. Therefore, it is suggested that the modifier pellet content not exceed 20% of the asphalt mass, unless some measures are taken to reduce viscosity in order to further increase the modifier pellet content. The viscosity of SBS I-D is between 10% Pellets MA and 20% Pellets MA.

The Arrhenius equation is widely employed to characterize the viscosity–temperature relationship of asphalt, as shown in Equations (1) and (2) [38].

(1) $$\eta =A\;⦁e^{E_{\eta }/RT}$$

(2) $$\ln\eta =\ln A+\frac{E_{\eta }}{R}\times \frac{1}{T}$$

where the viscosity of asphalt, represented by η, is defined in the equation, where A is a regression coefficient, R is the universal gas constant (8.314 J·mol^−1^·K^−1^), and T represents the absolute temperature, and the parameter Eη represents the flow activation energy. The straight line and related parameters of viscosity temperature fitting are shown in Figure 6b and Table 2. From the goodness of fit, it can be seen that R^2^ is above 0.97, which means that the Arrhenius equation can accurately describe the viscosity–temperature relationship of asphalt. In addition, further calculations can obtain the Eη of all asphalt. A lower Eη value indicates that asphalt flows more readily and exhibits greater temperature sensitivity [39,40]. From Table 2, it can be seen that adding modifier pellets can reduce the fluidity and temperature sensitivity of asphalt.

### 3.2. Rheological Properties

**Figure 7:**
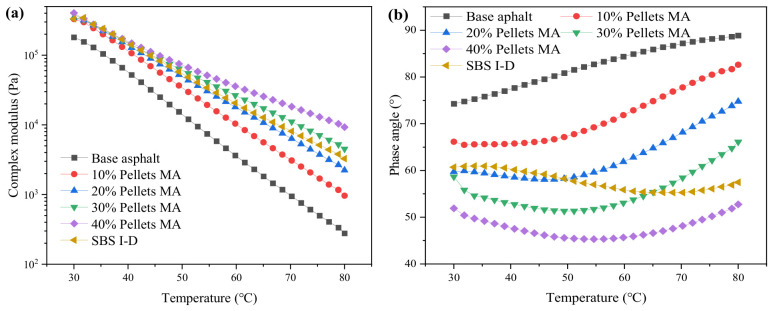
The result of temperature sweep of the original asphalt: (**a**) G*; (**b**) δ.

By conducting temperature sweep tests on base asphalt, modified asphalt with four different amounts of modifier pellets and SBS I-D, the relationship curves of complex modulus (G*) and phase angle (δ) with temperature can be obtained, as illustrated in Figure 7. G* is the overall indicator for measuring the deformation resistance of asphalt, with higher values corresponding to greater deformation resistance [41]. δ reflects the ratio of the viscous component to the elastic component of asphalt. The smaller the δ, the more significant the elastic properties of asphalt, and vice versa [42]. As observed in Figure 7a, the G* of all asphalt samples declines as temperature rises, indicating that the asphalt has weaker resistance to deformation under high temperature conditions. The incorporation of CR/SBS pellets increases the G* of asphalt, improves its resistance to deformation, and, with the increase in CR/SBS pellets dosage, the G* shows an increasing trend. The G* of SBS I-D is between 20% Pellets MA and 30% Pellets MA. From Figure 7b, the incorporation of CR/SBS pellets lowers the δ and enhances the elastic response of asphalt. These effects become more pronounced with higher pellet content, leading to a further reduction in δ and a greater proportion of elastic behavior. In addition, it was found that the δ of base asphalt gradually increased with temperature. The modified asphalt with four different pellet contents remained basically unchanged at 30 °C~50 °C, and the δ increased significantly after 50 °C. However, the phase angle of SBS I-D remained basically unchanged at 30 °C~65 °C and began to increase after 65 °C. This can be mainly explained by the progressive softening of the base asphalt as temperature increases, which increases the viscosity component and leads to a continuous increase in phase angle (close to 90°). However, adding modified pellets to asphalt changed this behavior, as the crumb rubber formed an elastic network structure in asphalt, which still provided sufficient elasticity after base asphalt softened, balanced the viscous flow trend, and stabilized the phase angle.

**Figure 8:**
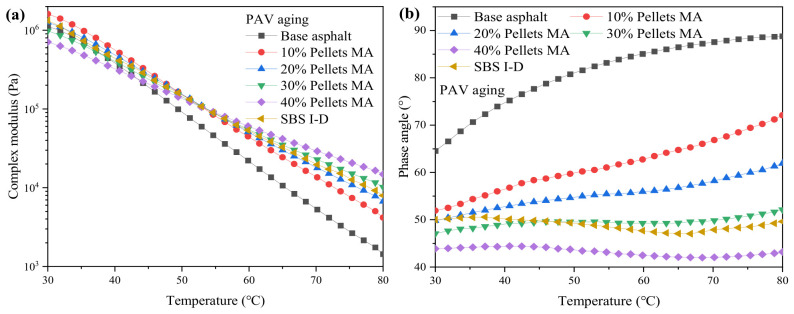
Temperature sweep results for the PAV-aged asphalt: (**a**) G*; (**b**) δ.

The G* and δ after PAV aging are presented in Figure 8. After PAV aging, the G* of all asphalt has increased and the δ has decreased. This occurs primarily because of the volatilization of light components or their conversion into heavy components during asphalt aging, resulting in a decrease in the proportion of light components and a corresponding rise in heavy components [43]. Moreover, the G* of asphalt modified with different dosages of modifiers intersected at around 50 °C, and at 30~50 °C, the lower the content of modifier, the larger the G* after aging. At 50~80 °C, although the trend of complex modulus changes in different asphalt samples is consistent with that of unaged asphalt samples, there is a varying degree of increase after aging.

**Figure 9:**
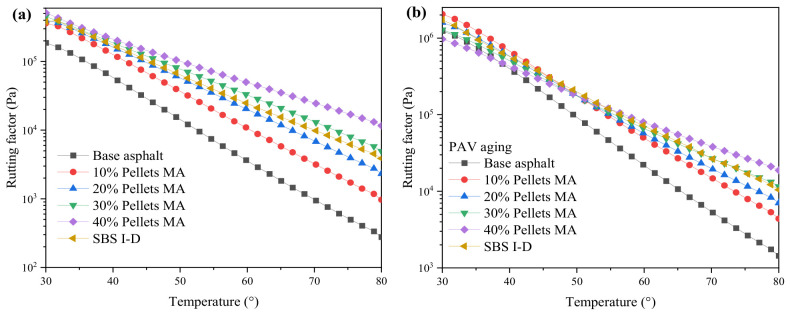
Rutting factor: (**a**) unaged asphalt; (**b**) aged asphalt.

The rutting factor (G*/sinδ), defined as the ratio of G* to sinδ, serves as a key indicator of asphalt’s high-temperature rutting resistance, with higher values corresponding to superior performance [44]. Through further calculation, Figure 9 presents the rutting factor of asphalt in both unaged and aged conditions. With increasing temperature, the rutting factor of asphalt progressively declines, indicating a reduction in its resistance to high-temperature deformation. This is also the main reason why asphalt pavements are prone to rutting in summer when the temperature is higher. In addition, under the same temperature conditions, a higher content of CR/SBS pellets leads to an increased rutting factor in unaged asphalt, reflecting enhanced deformation resistance under high-temperature conditions. The rutting factor of unaged asphalt does not cross within the measured temperature range (30 °C~80 °C), while the rutting factor of SBS I-D is between 20% Pellets MA and 30% Pellets MA. In comparison to unaged asphalt, the rutting factor of all aged asphalt has increased, but the degree of increase varies.

**Figure 10:**
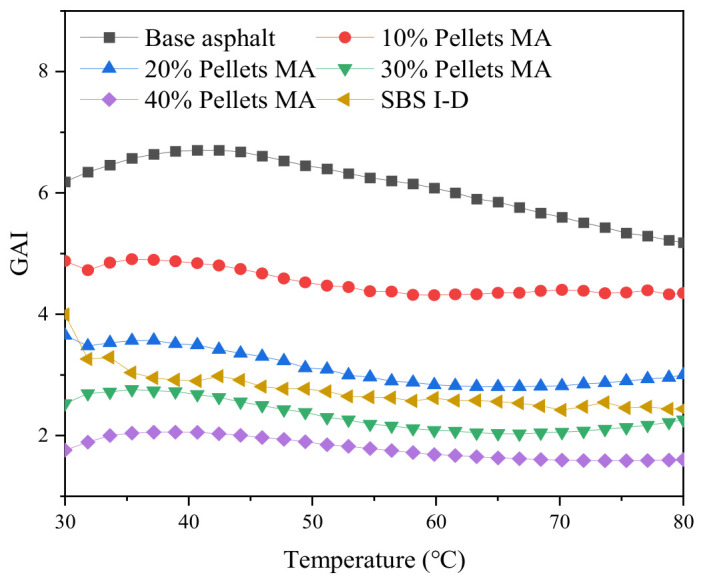
The GAI of asphalt samples.

Given the inconsistent increase in complex modulus before and after aging, researchers have proposed using the ratio of aged to unaged G* as an indicator for evaluating asphalt aging resistance [45]. A higher ratio indicates a more pronounced increase in G* after aging, corresponding to poorer anti-aging performance of the asphalt. The complex modulus aging index (GAI), defined as the ratio of the G* of aged asphalt to that of unaged asphalt, is presented in Figure 10. The GAI of asphalt fluctuates to some extent at different temperatures, but the differences in GAI between different asphalt types are quite significant. It was found that at the same temperature, the GAI of base asphalt was the highest, which means that the anti-aging performance of base asphalt is the worst. Furthermore, the GAI of modified asphalt progressively decreases with increasing modifier content. These results demonstrate that the addition of CR/SBS pellets enhances the aging resistance of asphalt. The anti-aging performance of SBS I-D is between 20% Pellets MA and 30% Pellets MA.

**Figure 11:**
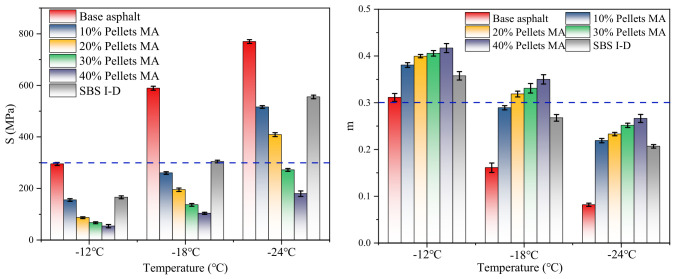
Creep stiffness (S) and creep rate (m) at 60 s of BBR test.

The bending beam rheometer (BBR) test serves as an effective method for assessing the low-temperature performance of asphalt [46]. Figure 11 presents the creep stiffness (S) and creep rate (m) of six types of asphalt at 60 s, with lower S values and higher m values indicating excellent low-temperature performance of the asphalt. SHRP requires the S and m at 60 s, with S ≤ 300 and m ≥ 0.3 [47]. With decreasing temperature, all asphalt samples exhibit an increase in S and a decrease in m. Base asphalt demonstrates the poorest low-temperature performance, whereas the CR/SBS pellet-modified asphalt presents a clear enhancement in low-temperature performance as the pellet content rises. At −12 °C, all asphalt samples satisfied the SHRP specification requirements. Nonetheless, at −18 °C, the base asphalt failed to meet the criteria, while the four pellet-modified asphalts and SBS I-D remained compliant. At the lower temperature of −24 °C, none of the asphalt samples fulfilled the specification requirements.

### 3.3. FTIR

**Figure 12:**
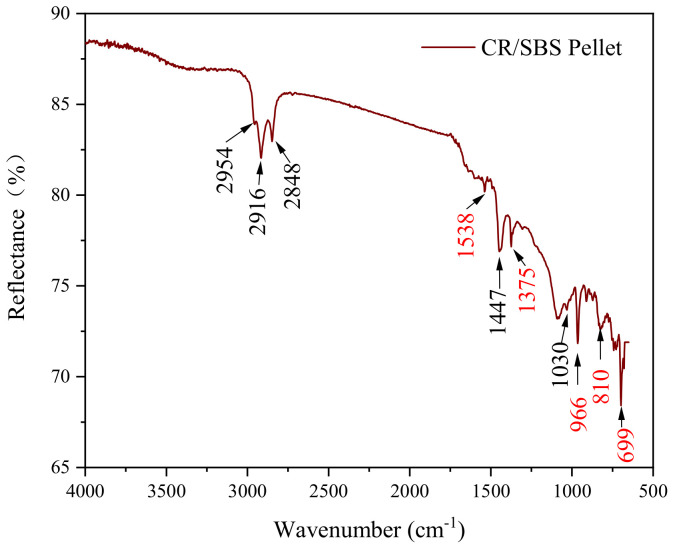
The FTIR spectrum of CR/SBS pellets.

The infrared spectrum of CR/SBS pellets is shown in Figure 12. Absorption peaks with distinct intensities appear at different wavenumbers, and their assigned functional groups are summarized in Table 3. From the figure, the absorption peaks at 1538 cm^−1^, 1375 cm^−1^ and 810 cm^−1^ are characteristic peaks of natural rubber [48]. The absorption peaks at 966 cm^−1^ and 699 cm^−1^ are formed by double bonds in polybutadiene and benzene rings in polystyrene, respectively, indicating the presence of SBS or SBR in the CR/SBS pellet composition [49]. This also indicates that the main material composition of modifier pellets is composed of CR and SBS.

**Figure 13:**
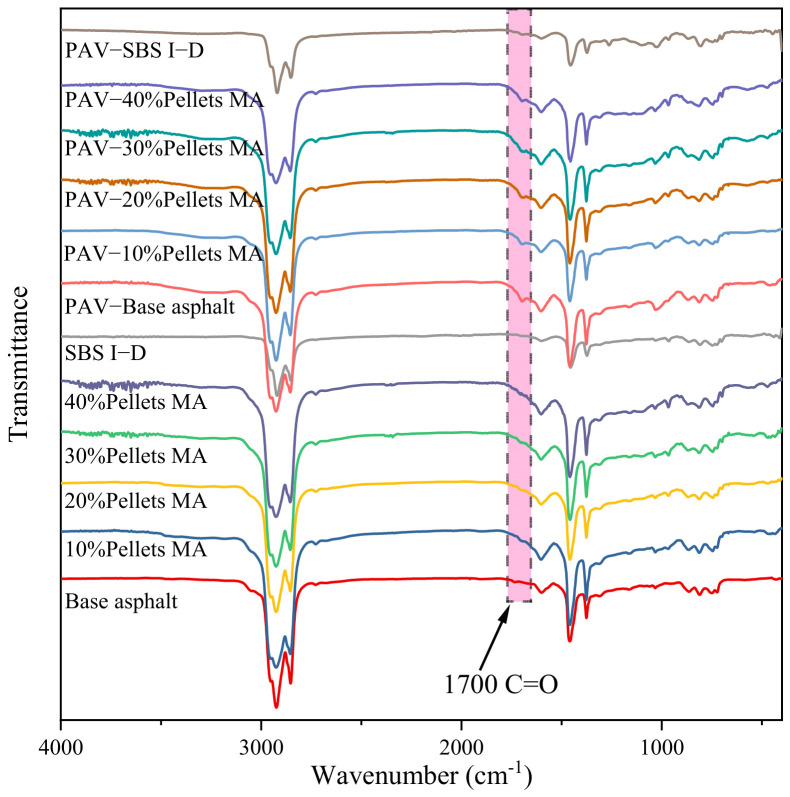
The infrared spectrum of asphalt.

**Figure 14:**
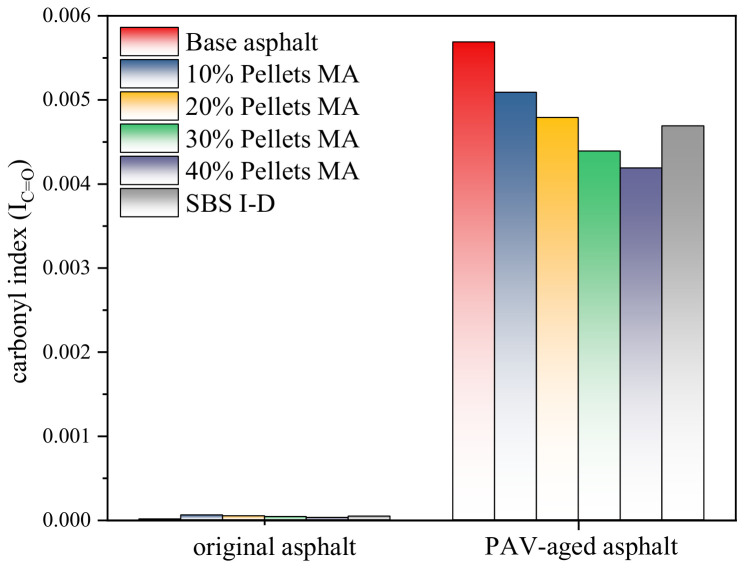
I_C=O_ of unaged asphalt and PAV-aged asphalt.

In addition, the FTIR spectrum of base asphalt, asphalt modified with CR/SBS pellets, SBS I-D, and their asphalt after PAV aging were tested, as shown in Figure 13. Compared with base asphalt, it was found that no new characteristic peaks appeared in the infrared spectra of CR/SBS pellet-modified asphalt and SBS I-D. This finding further suggests that the modification mechanism of CR/SBS-polymerized pellets in asphalt is predominantly physical blending, which aligns with previously reported results in the literature [32,52]. Previous studies have shown that the carbonyl index (I_C=O_) in asphalt increases after aging, and the value increases with the degree of aging [53]. To further assess the aging resistance of CR/SBS pellet-modified asphalt with varying pellet contents and SBS I-D, the peak area at 1700 cm^−1^ and the total integrated area in the spectral region of 600–2000 cm^−1^ were calculated using FTIR processing software (OMNIC8.2). I_C=O_ is equal to the ratio of the area of the absorption peak at 1700 cm^−1^ to the total area of the absorption peak in the range of 600~2000 cm^−1^. The I_C=O_ calculation results of all asphalt samples are presented in Figure 14. The I_C=O_ of the original asphalt is very small, and the difference between different asphalts is not significant. However, the I_C=O_ of the CR-/SBS-modified asphalt is slightly higher than that of the base asphalt, which may be due to a certain degree of aging of the asphalt during the modification process. After PAV aging, the I_C=O_ of all asphalts increased significantly, and the I_C=O_ of base asphalt was the highest, indicating that the aging-resistant performance of base asphalt was the worst. With the increase in CR/SBS pellets dosage, the I_C=O_ of CR/SBS pellet-modified asphalt shows a decreasing trend. This phenomenon can likely be due to the presence of aging inhibitors in waste tire rubber powder. The I_C=O_ of SBS I-D is between 20% pellets MA and 30% pellets MA, which is consistent with the conclusion drawn from the GAI.

### 3.4. Thermal Stability

**Figure 15:**
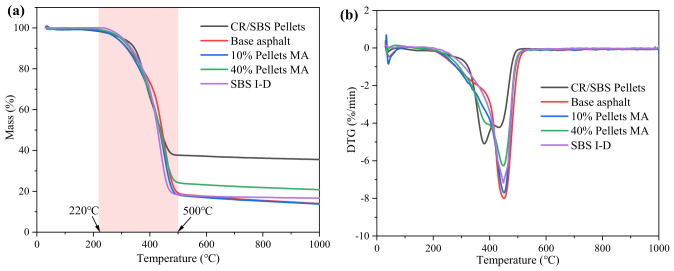
Results of thermogravimetric analysis: (**a**) TG curve; (**b**) DTG curve.

Thermal stability tests were conducted on CR/SBS pellets, base asphalt, 10% pellets MA, 40% pellets MA and SBS I-D using a differential scanning calorimeter. The test results are presented in Figure 15. As observed in Figure 15a, both the CR/SBS pellets and the asphalt samples gradually decompose with increasing temperature and eventually stabilize. The CR/SBS pellet and all asphalt samples have almost no mass loss from room temperature to 220 °C, undergo significant thermal decomposition from 220 °C to 500 °C, and tend to stabilize after 500 °C. Figure 15b shows the relationship between decomposition rate and temperature. The DTG curves of both the CR/SBS pellets and the modified asphalt with higher pellet content exhibit two distinct peaks, indicating that there are mainly two substances in the modified agent particles and high-dose modified asphalt, and their pyrolysis rates are inconsistent. However, matrix asphalt, low-content modifier-modified asphalt, and SBS only have one peak. In addition, the order of pyrolysis rate is base asphalt > 10% pellets MA > 40% pellets MA > CR/SBS pellets, and the order of residual mass is CR/SBS pellets > 40% pellets MA > 10% pellets MA > base asphalt.

### 3.5. Phase Morphology

**Figure 16:**
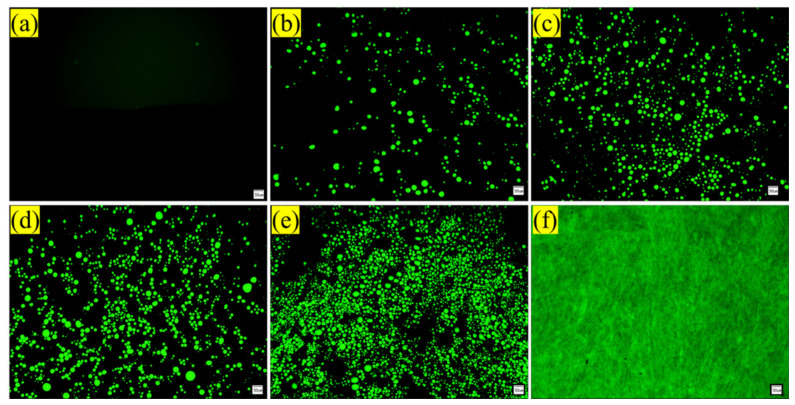
Fluorescence image: (**a**) base asphalt; (**b**) 10% pellets MA; (**c**) 20% pellets MA; (**d**) 30% pellets MA; (**e**) 40% pellets MA; (**f**) SBS I-D.

Figure 16 presents fluorescent images of CR/SBS pellets, base asphalt, modified asphalt with CR/SBS pellets, and SBS I-D. Consistent with the prior literature, base asphalt appears black under observation and exhibits no fluorescence [54]. With the increase in CR/SBS pellets dosage, there are more bright spots in the pellet-modified asphalt. In addition, due to the extremely low content in the CR/SBS pellets composition, SBS did not exhibit significant fluorescence effects, and the bright spots in the CR/SBS pellet-modified asphalt were mainly generated by crumb rubber. The fluorescence images of SBS I-D and pellet-modified asphalt are significantly different. In SBS I-D, the SBS modifier and asphalt are completely integrated, and it is no longer possible to distinguish between SBS and asphalt. However, in CR/SBS pellet-modified asphalt, CR and asphalt are not integrated, and CR is distributed in particles in the asphalt. This also represents a key factor contributing to the performance differences between pellet-modified asphalt and SBS I-D.

## 4. Conclusions

This paper uses SBS-modified asphalt (SBS I-D) as the control group and evaluates the physical characteristics, rheological behavior, thermal stability, and modified mechanism of pellet-modified asphalt through a series of experimental studies. The conclusion is as follows:

(1) Incorporating CR/SBS pellets into base asphalt reduces penetration while increasing softening point and viscosity. Higher pellet content leads to progressively lower penetration and further elevation in softening point and viscosity. The penetration and viscosity of SBS I-D are between 10% pellets MA and 20% pellets MA, and the softening point is between 20% and 30% pellets MA.
(2) Compared with base asphalt, CR/SBS pellet-modified asphalt has significantly improved high-temperature and low-temperature performance. The rutting factor of SBS I-D is between 20% pellets MA and 30% pellets MA, and its low-temperature crack resistance is similar to that of 10% pellets MA.
(3) The infrared spectroscopy results indicate that no new functional groups have appeared in the CR/SBS pellet-modified asphalt, further suggesting that the particle-modified asphalt is a physical mixing process.
(4) The aging resistance of the modified asphalt improves progressively as the content of CR/SBS pellets increases. The GAI shows that the anti-aging performance of SBS I-D is between 20% pellets MA and 30% pellets MA. This trend aligns with the variation pattern observed for the carbonyl index in the FTIR spectra.
(5) The thermal stability analysis results indicate that the pyrolysis temperature range of the modifier pellets and asphalt samples is between 220 °C and 500 °C. The order of pyrolysis rate is base asphalt > 10% pellets MA > 40% pellets MA > CR/SBS pellets, and the order of residual mass is CR/SBS pellets > 40% pellets MA > 10% pellets MA > base asphalt.
(6) The fluorescence images indicate that the pellet modifier in the CR/SBS pellet-modified asphalt is distributed in dots in the asphalt, while the SBS modifier is integrated with the base asphalt in the SBS I-D.

This study systematically investigates the performance and modification mechanism of CR/SBS pellet-modified asphalt. The results indicate that when the modifier content is 20%, the comprehensive performance of the modified asphalt reaches a level comparable to that of SBS-modified asphalts. Therefore, this dosage is recommended for practical engineering applications. On this basis, future research will focus on further increasing the dosage of modifiers to achieve higher performance, while systematically assessing the environmental impact and carbon footprint of the modifier, so as to provide a more solid theoretical foundation for the widespread application of CR/SBS pellets.

## Figures and Tables

**Table 1:** The technical index of asphalt.

Indexes	Base Asphalt		SBS I-D		Specification
	Test Results	Requirements	Test Results	Requirements	
25 °C penetration/0.1 mm	68.6	60~80	51.6	30~60	T0604
Softening point/°C	48.4	≥46	68.7	≥60.0	T0606
Ductility/cm	185	≥100 (15 °C)	23.5	≥20 (5 °C)	T0605
Viscosity/Pa·s	0.41 (135 °C)	/	2.46	≤3 (135 °C)	T0625

**Table 2:** Relevant parameters for fitting the Arrhenius equation.

Asphalt	Fitting Equation	E_η_ (kJ/mol)	Goodness of Fit R^2^
Base asphalt	lnη=6718.3/T−17.3	55.9	0.988
10% Pellets MA	lnη=7327.1/T−17.7	60.9	0.990
20% Pellets MA	lnη=7475.2/T−17.0	62.1	0.975
30% Pellets MA	lnη=7786.3/T−16.6	64.7	0.998
40% Pellets MA	lnη=8142.5/T−16.3	67.7	0.999
SBS I-D	lnη=7639.3/T−17.8	63.5	0.999

**Table 3:** Functional groups corresponding to absorption peaks.

Wave Numbers (cm^−1^)	Functional Groups and Vibration Type
2954	Asymmetric stretching vibration of −CH_3_
2916	Asymmetric stretching vibration of −CH_2_-
2848	Symmetric stretching vibration of −CH_2_-
1538	Nitrogen-containing functional group
1447	The superposition of the antisymmetric angular vibration of −CH_3_ and the in-plane angular vibration of −CH_2_
1375	Symmetric angular vibration of CH_3_ [50]
1030	Sulfoxide (>S=O) [51]
966	−C=C− of polybutyl diene segment
810	Cis−substituted C-H bond out-of-plane bending vibration [50]
699	C−H stretching vibration from the benzene ring in the polystyrene segment

## Data Availability

The raw data supporting the conclusions of this article will be made available by the authors on request.

## Abbreviations

The following abbreviations are used in this research:

CR	Crumb rubber
SBS	Styrene–butadiene–styrene block copolymer
FTIR	Fourier transform infra-22 red spectroscopy
FM	Fluorescence microscopy
DSR	Dynamic shear rheometer
BBR	Bending beam rheometer
DSC	Differential scanning calorimetry
G*	Complex modulus
δ	Phase angle
G*/sinδ	Rutting factor
GAI	Complex modulus aging index
S	Creep stiffness
m	Creep rate
PAV	Pressure aging vessel
I_C=O_	Carbonyl index
TG	Thermogravimetry
DTG	Derivative thermogravimetry
